# Analysis of the Current Situation and Influencing Factors of Clinical Nurse Association Standard Evidence-Based Practice

**DOI:** 10.1155/jonm/3313404

**Published:** 2025-05-27

**Authors:** Meng Xiao, Tao Zhou, Yanling Pei

**Affiliations:** Nursing Department, China-Japan Union Hospital of Jilin University, Changchun, Jilin, China

**Keywords:** association standard, Chinese Nursing Association standard, evidence-based nursing practice, evidence-based practice, nurse

## Abstract

**Aim:** The aim of this study was to investigate the current situation of clinical nurses with regard to association standard evidence-based practice (EBP) of the Chinese Nursing Association, analyze the factors affecting clinical nurses in carrying out association standard EBP, and provide scientific basis for further improving the level of clinical nurse association standard EBP.

**Design:** An observational, cross-sectional study design.

**Participants:** A total of 225 nurses in the departments of neurology and neurosurgery of a tertiary hospital in the Jilin province of China.

**Methods:** The questionnaire survey was conducted using the general data questionnaire, the standard EBP cognition scale of clinical nurses, the standard EBP belief scale of clinical nurses, the standard EBP behavior scale of clinical nurses, and the organizational atmosphere scale of clinical nurses.

**Results:** Clinical nurse association standard EBP cognition score was 26.66 ± 5.22 points, belief score was 17.92 ± 3.50 points, and behavior score was 31.08 ± 5.10 points. Multiple linear regression analysis showed that organizational atmosphere was the main factor affecting the cognition of evidence-based nursing practice of clinical nurses (*p* < 0.05). Scientific research experience and organizational atmosphere were the main influencing factors of clinical nurses' belief in evidence-based nursing practice (*p* < 0.05). The degree of understanding of nursing association standards related to the department, cognition of association standard, and organizational atmosphere were the main influencing factors of clinical nurses' evidence-based nursing practice behavior (*p* < 0.05).

**Conclusions:** The study reveals that clinical nurses demonstrate high levels of cognition, belief, and behavior regarding association standard EBP. Key influencing factors include organizational atmosphere, scientific research experience, and understanding of association standards. Nursing managers should foster a supportive work environment, leverage research talents, and organize comprehensive training to enhance evidence-based nursing practice further.

## 1. Introduction

Evidence-based practice (EBP) is a method that combines scientific research with specific clinical practice [1]. It is widely recognized as the gold standard for delivering high-quality, safe, and effective patient care [2]. Relevant studies have shown that EBP based on association standard in clinical nursing work can standardize nursing behavior, improve the quality of nursing decision-making, and improve patient health outcomes [3, 4]. However, despite its importance, EBP is not the standard of care globally, and there are still gaps in its implementation in clinical settings [5]. The patients admitted to the neurology and neurosurgery departments are in a critical condition, have many complications, stay for a long time, and have many basic nursing and specialized nursing operations, which are difficult and demanding. Among the association standards released by the Chinese Nursing Association, 22 association standards such as “Intravenous Thrombolysis Nursing For Patients With Ischemic Stroke” and “Feeding Nursing Of Stroke Patients With Dysphagia” can be promoted and applied in the neurology and neurosurgery department. These standards provide a valuable framework for nursing practice and have the potential to significantly improve patient outcomes. At present, most Chinese researches focus on the investigation of the implementation of a single association standard, and there is a lack of systematic and comprehensive research on the status quo of EBP and influencing factors based on association standards. This gap in the Kumah's literature highlights the need for a more in-depth and holistic analysis of the current situation and factors influencing the implementation of EBP based on association standards in clinical nursing practice [5]. Therefore, by understanding the current situation of EBP of association standard for neurology and neurosurgery departments' nurses and analyzing the influencing factor, this study provides reference for further implementation of association standard in the future. This research will fill the gap in the literature and contribute to the development of strategies to enhance the adoption and effectiveness of EBP in clinical nursing practice.

## 2. Background

Association standard as a standard document was independently formulated by social organizations, playing a positive leading role in promoting the standardized operation of the industry, improving the quality of industry management, and promoting industry innovation [6]. In 2017, the Standardization Law of the People's Republic of China officially clarified the legal status of association standard in the China standards system [7]. In 2022, the opinions on promoting the high-quality development of association standard emphasize that it is necessary to broaden the application channels of association standard and deepen the knowledge and understanding of association standard [8]. Along with the development of health in China, nursing needs to further promote the standardization construction. As an important part of technical standards, nursing association standard emerges. After the establishment of the Standards Working Committee of the Chinese Nursing Association, 40 association standards such as “Cancer Pain Management in Adults Patients” have been issued since 2019 [9], which have played a positive role in standardizing and leading the development of nursing professionalism and provided evidence-based support for standardized nursing practice operations.

## 3. Methods

### 3.1. Aim

#### 3.1.1. Primary Objective

To investigate the current situation of clinical nurse association standard EBP in the Chinese Nursing Association. This includes assessing the extent to which clinical nurses in neurology and neurosurgery departments are implementing association standards in their daily practice.

#### 3.1.2. Secondary Objectives

To identify and analyze the factors influencing clinical nurses' implementation of association standard EBP. This involves examining individual, organizational, and contextual factors that may act as barriers or facilitators to the adoption of EBPs.

To provide a scientific basis for developing strategies to improve the level of clinical nurse association standard EBP. By understanding the current situation and influencing factors, this study aims to offer actionable recommendations for enhancing the integration of EBPs into clinical nursing.

#### 3.1.3. Research Focus and Expected Outcomes

Current Implementation Status: A comprehensive assessment of how well clinical nurses are adhering to the association standards in EBP.

Influencing Factors: An in-depth analysis of the factors affecting the implementation of EBP, including individual nurse characteristics, organizational support, and external environment.

Strategic Recommendations: Based on the findings, the study will propose targeted interventions and strategies to bridge the gap between evidence and practice, ultimately improving patient care quality.

### 3.2. Study Design

A cross-sectional survey design was used.

### 3.3. Sample and Setting

A total of 225 nurses from the Department of Neurology and the Department of Neurosurgery of a tertiary hospital in the Jilin province of China were selected as the survey objects by convenience sampling method in February 2024. Participants were required to meet the following for study eligibility: active registered nurses who are currently working in the Department of Neurology or Department of Neurosurgery for at least 6 months. Nurses who are undergoing standardized training, nurses who had been on vacation for more than 3 months in the 6 months before the survey began, and nurses who refused to participate in this survey were excluded. According to the Kendall sample size criterion, the sample size is 5–10 times the number of questionnaire items [10]. Considering invalid questionnaires in sample recovery, the sample size is expanded by 10%. The questionnaire on the status of clinical nurse association standard EBP included 24 observational variables, with a sample size of at least 132 cases. A total of 231 questionnaires were sent out in this study, and 225 questionnaires were effectively recovered, with an effective recovery rate of 97.40%. To ensure that ethical standards were met, the following informed consent procedures were implemented: The head nurses provided a detailed explanation to the nurses about the purpose of the study, the voluntary nature of participation, and the confidentiality of their responses. It was clearly communicated that participants had the right to withdraw from the study at any time without any penalty or loss of benefits. The information was presented in a clear and understandable manner to ensure that participants fully understood the study's objectives and procedures. Nurses were given ample time to ask questions and seek clarification before deciding to participate. In addition, participants were required to acknowledge their understanding of the study information through an electronic consent form embedded in the questionnaire. All participants agreed to participate in the study voluntarily. This study has been approved by the hospital ethics committee (2024112106).

### 3.4. Instruments

#### 3.4.1. Clinical Nurse Association Standard EBP Questionnaire

According to the purpose of the study, the questionnaire was prepared by literature review [11–13], combined with research group discussion and expert correspondence. Two rounds of correspondence were conducted with 15 experts in the field of nursing management and clinical nursing. The recovery rates of the two rounds of correspondence questionnaires were 100%, and Kendall harmony coefficients were 0.394 and 0.463, respectively. The Kendall harmony coefficients of all indicators were statistically significant (*p* < 0.05), and experts' evaluation of the questionnaire was consistent. The authority coefficient of experts is 0.89–0.93, and the authority degree of experts is high. Finally, a questionnaire on the current situation of clinical nurse association standard EBP was formed, with Cronbach's α coefficient of 0.981. The questionnaire consisted of five aspects: (1) General data questionnaire, including gender, age, department, years of work, etc. (2) Clinical nurse association standard EBP cognition scale: A total of six items, using the Likert five-level scoring method, ranging from “very inconsistent” to “very consistent” of 1–5 points, respectively, with a total score of 30 points. The higher the score, the better the clinical nurse association standard EBP cognition. Cronbach's α coefficient of this scale is 0.989. The structural validity was tested by the SPSS principal component analysis and maximum variance rotation method through exploring factor analysis (EFA). The results showed that the Kaiser–Meyer–Olkin (KMO) test statistic was 0.932, and Bartlett's sphericity test *χ*^2^ = 2856.727, *p* < 0.001. The total variance was interpreted as 94.650%, and the factor loads of the six observed variables were all greater than 0.500, indicating that the questionnaire had good structural validity. (3) Clinical nurse association standard EBP belief scale: A total of four items, using Likert five-level scoring method, ranging from “very inconsistent” to “very consistent” of 1–5 points, respectively, with a total score of 20 points. The higher the score, the stronger the clinical nurse association standard EBP belief. Cronbach's α coefficient of this scale is 0.994. The KMO test statistic was 0.846; Bartlett's sphericity test *χ*^2^ = 2272.227, *p* < 0.001; the total variance interpretation was 98.332%; and the factor loads of the four observed variables were all greater than 0.500, indicating that the questionnaire had good structural validity. (4) Clinical nurse association standard EBP behavior scale: A total of five items, using the Likert seven-level scoring method, from “never” to “often” of 1–7 points, respectively, with the total score of 35 points. The higher the score, the better the clinical nurse association standard EBP behavior. Cronbach's α coefficient of this scale is 0.964. The KMO test statistic was 0.906; the Bartlett's sphericity test *χ*^2^ = 1377.430, *p* < 0.001; the total variance interpretation was 87.703%; and the factor load of the five observed variables were all greater than 0.500, indicating that the questionnaire had good structural validity. (5) Organizational atmosphere of clinical nurse association standard EBP: The scale was divided into 25 items in 7 dimensions, including organizational philosophy, working style, environmental atmosphere, teamwork, leadership effectiveness, learning and growth, and resource provision. Likert five-level scoring method was adopted, ranging from “very inconsistent” to “very consistent,” with a total score of 125 points. The higher the score, the better the organizational atmosphere of clinical nurse association standard EBP. Cronbach's α coefficient of this scale is 0.990. The KMO test statistic was 0.943, *χ*^2^ = 12,402.437 of Bartlett's sphericity test, *p* < 0.001; the total variance interpretation was 85.310%; and the factor load of 25 observed variables were all greater than 0.500, indicating that the questionnaire had good structural validity.

### 3.5. Data Collection

In February 2024, the researcher contacted the head nurses of each ward of the departments of neurology and neurosurgery to communicate and explain the survey purpose, survey objects, and questionnaire filling methods, and the head nurses sent the questionnaire to the nurses in the department to fill in through the questionnaire star platform. Participants responded anonymously using smart devices. The questionnaire instructions explain the purpose and significance of the research and the method of filling in the questionnaire. All questions are required. To prevent multiple responses on the same device, each IP address of a survey object can be answered only once. After the questionnaire is collected, the two persons check the data in time to ensure the quality of the questionnaire.

### 3.6. Data Analysis

The data were imported from the questionnaire into Excel table, and SPSS 25.0 statistical software was used to analyze the data. Qualitative data were described by number and component ratio (*n*, %). Quantitative data follow a normal distribution, described by mean ± standard deviation ($\bar{x}\pm s$). Independent sample *t*-test and single factor analysis of variance were performed. The variables with statistical significance in the single factor analysis were taken as independent variables and multiple linear regression analysis was used. A *p* value < 0.05 indicated statistical significance.

## 4. Results

### 4.1. Demographic Characteristics

Demographic characteristics are summarized in Table 1.

### 4.2. Clinical Nurse Association Standard EBP Cognition, Belief, and Behavior Scores

The overall score of clinical nurse association standard EBP cognition was 26.66 ± 5.22 points, the overall score of clinical nurse association standard EBP belief was 17.92 ± 3.50 points, and the overall score of clinical nurse association standard EBP behavior was 31.08 ± 5.10 points. The average score of each item is shown in Table 2.

### 4.3. Single Factor Analysis of the Status Quo of Clinical Nurse Association Standard EBP

Gender, teaching experience, understanding of nursing association standard relevant to department, whether or not have received training on association standard, and the degree of recognition of the nursing profession had statistically significant differences in the cognitive score of EBP of association standard (*p* < 0.05); gender, teaching experience, scientific research experience, whether or not have received training on association standard, and the degree of recognition of the nursing profession had statistically significant differences in association standard EBP belief score (*p* < 0.05); understanding of nursing association standard relevant to department, whether or not have received training on association standard, and the degree of recognition of the nursing profession had statistically significant differences in the behavior score of EBP of association standard (*p* < 0.05). Details are reported in Table 3.

### 4.4. Multiple Linear Regression Analysis of the Current Situation of EBP of Clinical Nurse Association Standard

The scores of cognition, belief, and behavior of association standard evidence-based nursing practice were taken as dependent variables, and the scores of demographic characteristics and organizational atmosphere that had statistical significance for cognition, belief, and behavior of evidence-based nursing practice in single factor analysis were taken as independent variables for multiple linear regression analysis. The assignment of independent variables was shown in Table 4.  Table 5 showed that the organizational atmosphere was the main factor affecting the cognition of evidence-based nursing practice of clinical nurses. Scientific research experience and organizational atmosphere were the main influencing factors of clinical nurses' belief in evidence-based nursing practice. The Understanding of nursing association standard relevant to department, cognition of association standard, and organizational atmosphere are the main influencing factors of clinical nurse evidence-based nursing practice. Details are reported in Table 5.

## 5. Discussion

### 5.1. Clinical Nurse Association Standard EBP Cognition, Belief, and Behavior Are at a High Level

The results of this study showed that clinical nurse association standard EBP cognition score was 26.66 ± 5.22 points, indicating that nurses had a good cognition of the relevant association standard of the department, which was similar to the results of recent research [14]. The results of Yuxia et al. [15] showed that clinical nurses had a moderate cognition level of group standards for incontinence dermatitis, which may be related to the complex condition and high nursing requirements of neurology patients, and nurses were more inclined to apply multiple group standards to improve nursing quality. This may be due to the promotion and popularization of association standard of the Chinese Nursing Association in recent years. A survey unit has continued to carry out the interpretation, training, and the knowledge competition of association standard, which helps to improve the cognition of the survey subjects. On the other hand, the degree structure of the respondents accounted for 88.9% of the undergraduate degree, and the higher educational experience may be the factor responsible for the improvement of nurses' cognition of association standard. Among the cognitive scale, a high score item is “I take the initiative to learn the association standard related to own department,” with a score of 4.47 ± 0.88. This indicates that nurses have the motivation and need for independent learning. The item with a low score was “I know the retrieval channels of association standard,” with a score of 4.39 ± 0.92, mainly because the current training focuses on the content and importance of group standards, and there is less training on how to efficiently and accurately search group standards, which makes it difficult for nurses to quickly find the required group standards in practical work. It indicates that in the future, it is necessary to cultivate nurses' ability to search association standard efficiently and accurately, so as to further improve their cognitive level and application ability.

The association standard EBP belief score of clinical nurses was 26.66 ± 5.22, similar to the results of Yaping and Danping [16]. In our investigation, most clinical nurses agreed to carry out association standard EBP in their department, possibly because the nursing department of our hospital regularly organizes association standard knowledge training to encourage and guide nurses to carry out EBP based on association standard in clinical work. Lijuan et al. [17] also showed that after training, the total score of the belief dimension for group standards increased significantly, which was mainly reflected in the increased sense of authority of group standards. This indicates that through systematic training, nurses can better understand the scientific and practical nature of group standards, and training plays an important role in improving nurses' trust and recognition of group standards. In particular, the score of “I am willing to continue to carry out association standard evidence-based nursing practice in my future work” was the highest, with a score of 4.49 ± 0.88, indicating that clinical nurses hope to improve their evidence-based ability and improve the quality of nursing practice in the long-term work stage, which has positive significance for the continuous promotion and application of association standard in clinical nursing practice.

The association standard EBP behavior score of clinical nurses in this study was 31.08 ± 5.10, which was at a high level, similar to the results of other domestic studies. Zhou et al. [18] investigated the status quo of nurses implementing nursing measures according to the association standard of “Nursing Care For Adult Patients With Oxygen Therapy,” and found that clinical nurse nursing behavior of oxygen therapy was better. Deng [19] conducted a survey and study on the implementation of the association standard of “Nursing Care Of Adult Patients With Enteral Nutrition Support” among 526 nurses, and the results showed that the implementation of the association standard was generally good. These similar research results show that different group standards have achieved positive results in the promotion and application of group standards. However, although different studies have shown a higher level of group standard practice application behavior, this study further analyzed the level of multiple group standard practice application behaviors affecting nurses in the neurology department. On the behavior scale, the item “Before implementing care, I translate the care questions to specific, clear, and scientific questions” got a high score of 6.29 ± 1.00, indicating that nurses can take the initiative to conduct evidence-based association standard before implementing nursing plans and nursing operations to ensure that nursing behaviors conform to nursing norms and standards. The low score was “I will evaluate the effectiveness of association standard practice,” with a score of 6.17 ± 1.10, indicating that nurses lacked the final summary evaluation of the implementation effect. The reasons are as follows: some nurses lack the consciousness of summary evaluation and do not realize the importance of evaluating the effect of group standard practice; the lack of evaluation methods and tools in clinical work, such as the lack of standardized evaluation indicators, evaluation procedures, and related training; nurses in the Department of Neurology have heavy work burden and high work intensity, and they may not have enough time and energy to evaluate the effect in the face of complex patient conditions and intense nursing tasks. The lack of incentive mechanism in the department leads to the lack of evaluation of enthusiasm of nurses. The core of evaluation is to compare whether and to what extent the selected group standard can improve the nursing effect of patients. Therefore, managers should further improve nurses' awareness of group standard practice effect evaluation through systematic training and publicity. Construct the evaluation system of group standard practice application effect, and guide nurses to apply it correctly; at the same time, the nursing process is optimized, the work tasks are rationally allocated, and enough time and energy are provided for nurses to evaluate the effect. An incentive mechanism is also established to encourage nurses to actively participate in the evaluation of the effect of group standard practice, such as the establishment of a reward system and the inclusion of performance appraisal, so as to ensure the continuity and standardization of the application of group standard practice.

### 5.2. The Influencing Factors of Clinical Nurse Association Standard EBP

#### 5.2.1. Organizational Atmosphere

A good organizational atmosphere is one of the key factors affecting the effective implementation of EBP. The results of this study showed that the organizational atmosphere score was 113.89 ± 15.54, which was at a high level. The results of multiple factors showed that the organizational atmosphere was the common factor affecting the cognition, belief, and behavior of the association standard EBP of the clinical nurse. The higher the score of the organizational atmosphere, the higher the score of the association standard EBP cognition, belief, and behavior of the clinical nurse. William et al. [20] showed that when the organizational atmosphere is good, the frequency of doctors using EBP will increase, and this study also produced consistent results for nurses. This further confirms the general importance of organizational climate in different medical occupational groups. Positive organizational atmosphere is crucial to promote clinical nurses' participation in EBP, which can increase their enthusiasm for EBP and promote clinical nurses to participate in association standard EBP more actively. This suggests that nursing managers should provide nurses with a supportive environment, and clinical nurses should develop a clear association standard to guide EBP when accessing the resources provided by the organizational environment [21]. At the same time, cultivating team spirit, encouraging collaboration and knowledge sharing, and improving nurses' professional skills based on the association standard of the Chinese Nursing Association through continuous learning and training are all important strategies to promote clinical nurses to carry out association standard EBP, so that hospitals can effectively promote the development of association standard evidence-based nursing practice and improve the overall nursing level.

#### 5.2.2. Scientific Research Experience

Scientific research experience, as an important way to improve nurses' professional quality and skills, has a positive impact on improving nurses' attitude toward association standard EBP and playing a leading role. The results of this study show that scientific research experience is the main influencing factor of clinical nurse belief in association standard evidence-based nursing practice. EBP has a specific model and framework, including asking questions, obtaining the best evidence, applying evidence to clinical practice, and so on, which requires nurses to have the ability to find evidence and the insight to translate evidence into clinical practice [22]. Association standard evidence-based nursing practice steps are generation, promotion, and application of association standard in nursing practice, among which generation of association standard requires clinical nurses to be able to consult association standard related to the department, which requires clinical nurses to be able to propose clinical nursing problems and search association standard to solve clinical nursing problems, all of which are because of the ability of scientific research [23]. Therefore, nurses with scientific research experience showed a more positive attitude of association standard EBP under the atmosphere of attaching importance to evidence-based research. This study is similar to the findings of Mingyu et al. [24]. In their research, scientific research experience can affect the EBP of survey subjects. In order to further change nurses attitude toward association standard EBP in the future, it is imperative to optimize and adjust the clinical nurse team, give full play to the role of nurse with high education, senior and strong scientific research ability, and lead the general nurse to improve their willingness to use association standard. At the same time, nurses' scientific research ability can be improved through specific research promotion strategies (such as research training, research resource support, etc.), so as to further enhance their belief in the application of group standard practice.

#### 5.2.3. Understanding of Nursing Association Standard Relevant to Department and Cognition of Association Standard

The results of this study showed that understanding of nursing association standard relevant to department and cognition of association standard are the main influencing factors of clinical nurse evidence-based nursing practice behavior. The more they knew about relevant association standard, the higher their cognition level, the better the evidence-based atmosphere of the hospital/department, and the better the level of nurses' association standard EBP behavior. Xiaofang et al. [25] showed that understanding of evidence-based nursing was the main factor affecting the implementation of evidence-based nursing, which was consistent with the results of this study. For group standards, only when nurses fully understand their content, background, and importance can they actively apply these standards to guide nursing practice in clinical work. Cognitive differences may lead nurses to choose different nursing strategies and operation methods when facing complex clinical problems. Therefore, to improve nurses' understanding and cognition of group standards is the key to improve their practical application behavior. As the best practice benchmark in the nursing industry, the association standard issued by the Chinese Nursing Association gathers excellent practical experience in the nursing profession, having strong pertinence, practicality, and operability, and provides a model for clinical nursing work that can be used for reference. Nursing managers should ensure the implementation of association standard through various forms and ways, so that the association standard can fully play the role of improving the quality of nursing services. Therefore, managers should grasp the understanding and application level of clinical nurse knowledge of association standard EBP; carry out professional, scientific, and targeted training to improve clinical nurse mastery of association standard; and provide continuous supervision and feedback during clinical nursing practice to further standardize nursing operations that do not meet association standard to facilitate the effective integration of association standard into clinical practice.

## 6. Conclusions

The findings of this study have significant implications for clinical practice. By enhancing the organizational atmosphere and providing a supportive environment, nursing managers can create a culture that promotes the adoption of EBPs. This, in turn, can lead to improved patient outcomes, reduced medical errors, and enhanced overall quality of care. In addition, fostering scientific research experience among nurses can empower them to critically evaluate and apply the latest evidence in their practice, further enhancing the effectiveness of care. Moreover, ensuring that nurses have a thorough understanding of relevant association standards and providing continuous education and training can bridge the gap between evidence and practice, ultimately benefiting patients through more standardized and effective nursing interventions Therefore, the implementation of strategies to improve these factors can have a profound impact on clinical practice, leading to better patient care and outcomes. This study only preliminarily analyzed the influencing factors of the association standard EBP of clinical nurse through cross-sectional investigation. In the future, longitudinal studies may be considered to explore the longitudinal changes between the association standard EBP of clinical nurses and its influencing factors, so as to promote the application of the association standard of the Chinese Nursing Association in clinical nursing practice.

## 7. Limitations

There are still some limitations in this study. First of all, investigation was carried out in only one tertiary hospital in the Jilin Province. The sample size can be expanded in the future to further improve the credibility of the results. Secondly, the standards for reference application in the departments of neurology and neurosurgery are more consistent with the association standard of the Chinese Nursing Association, while the number of applications in other departments such as ophthalmology, thyroid surgery, and hand/foot surgery is relatively small. In order to avoid selection bias in the process of data collection, which could lead to deviation from the authenticity of the research results, the investigation was carried out only in the departments of neurology and neurosurgery.

## Figures and Tables

**Table 1:** Demographic characteristics (*N* = 225).

		*n*	%
Gender	Male	4	1.8
	Female	221	98.2

Age (years)	≤ 25	15	6.7
	26–30	42	18.7
	31–35	75	33.3
	36–40	55	24.4
	41–45	27	12.0
	46–50	4	1.8
	≥ 51	7	3.1

Department	Neurology	122	54.2
	Neurosurgery	103	45.8

Years of work (years)	≤ 5	49	21.8
	6–10	63	28.0
	11–15	63	28.0
	16–20	29	12.9
	≥ 21	21	9.3

Technical position	Nurse	108	48.0
	Senior nurse	58	25.8
	Supervisor nurse	54	24.0
	Associate professor of nursing	4	1.8
	Professor of nursing	1	0.4

Position	Nurse	217	96.4
	Head nurse	8	3.6

Education background	Junior college degree	25	11.1
	Bachelor degree	194	86.2
	Master degree or above	6	2.7

Teaching experience	Yes	186	82.7
	No	39	17.3

Scientific research experience	Yes	16	7.1
	No	209	92.9

Understanding of nursing association standard relevant to department	Grasp	119	52.9
	Know well	64	28.4
	Know	40	17.8
	Have not heard of	2	0.9

Whether or not have received training on association standard	Yes	198	88.0
	No	27	12.0

The degree of recognition of the nursing profession	Approve	210	93.3
	Normal	15	6.7
	Disagree	—	—

**Table 2:** Scores of cognition, belief, and behavior of clinical nurse association standard evidence-based practice (*N* = 225).

		Mean ± SD
Cognition	I am aware of the current association standard related to own department	4.45 ± 0.89
	I know the retrieval channels of association standard	4.39 ± 0.92
	I take the initiative to learn the association standard related to own department	4.47 ± 0.88
	I understand the content of the association standard related to own department	4.47 ± 0.87
	I grasp the emphasis of association standard related to own department	4.43 ± 0.91
	I understand the clinical significance of association standard	4.45 ± 0.89

Belief	I would like to try to carry out association standard evidence-based nursing practice	4.48 ± 0.89
	I would like to recommend association standard evidence-based nursing practice to my colleagues	4.48 ± 0.88
	I am willing to share association standard evidence-based nursing practice in academic exchanges	4.47 ± 0.89
	I am willing to continue to carry out association standard evidence-based nursing practice in my future work	4.49 ± 0.88

Behavior	Before implementing care, I translate the care questions to specific, clear, and scientific questions	6.29 ± 1.00
	Inquire about the relevant association standard based on the care issues raised	6.18 ± 1.10
	I will effectively integrate the obtained association standard with the clinic	6.20 ± 1.08
	I will evaluate the effectiveness of association standard practice	6.17 ± 1.10
	Share my association standard practice with colleagues	6.24 ± 1.16

Abbreviation: SD, standard deviation.

**Table 3:** Single factor analysis of cognition, belief, and behavior of clinical nurse association standard evidence-based practice (*N* = 225).

		Number (*n*)	Cognition (mean ± SD)	Belief (mean ± SD)	Behavior (mean ± SD)
Gender	Male	4	30.00	20.00	33.75 ± 2.50
	Female	221	26.60 ± 5.24^a^	17.89 ± 3.52^b^	31.04 ± 5.13

Age (years)	≤ 25	15	27.07 ± 3.69	18.47 ± 2.47	29.20 ± 4.72
	26–30	42	28.12 ± 3.22	18.52 ± 2.30	31.07 ± 5.53
	31–35	75	26.32 ± 5.37	17.77 ± 3.73	31.07 ± 4.47
	36–40	55	25.89 ± 5.96	17.35 ± 4.18	30.85 ± 5.71
	41–45	27	27.19 ± 5.22	18.11 ± 3.62	32.63 ± 4.13
	46–50	4	27.75 ± 2.87	18.50 ± 3.00	30.25 ± 9.50
	≥ 51	7	24.00 ± 9.17	18.29 ± 3.15	31.71 ± 5.53

Department	Neurology	122	26.70 ± 5.41	17.85 ± 3.82	31.34 ± 4.88
	Neurosurgery	103	26.60 ± 5.01	18.01 ± 3.09	30.78 ± 5.36

Years of work (years)	≤ 5	49	27.65 ± 3.58	18.35 ± 2.55	29.61 ± 5.43
	6–10	63	26.30 ± 5.14	17.76 ± 3.51	31.16 ± 5.34
	11–15	63	25.79 ± 6.65	17.24 ± 4.67	31.14 ± 4.87
	16–20	29	27.41 ± 3.26	18.59 ± 2.24	32.55 ± 3.88
	≥ 21	21	26.95 ± 5.85	18.57 ± 2.46	32.10 ± 5.32

Technical position	Nurse	108	26.67 ± 5.32	17.93 ± 3.67	31.33 ± 4.80
	Senior nurse	58	26.07 ± 5.92	17.34 ± 3.98	30.66 ± 5.45
	Supervisor nurse	54	27.07 ± 4.33	18.43 ± 2.58	30.78 ± 5.48
	Associate professor of nursing	4	28.50 ± 3.00	19.00 ± 2.00	33.75 ± 2.50
	Professor of nursing	1	30.00	20.00	35.00

Position	Nurse	217	26.60 ± 5.28	17.89 ± 3.55	31.03 ± 5.15
	Head nurse	8	28.25 ± 2.71	18.88 ± 1.81	32.50 ± 3.30

Education background	Junior college degree	25	26.56 ± 4.18	17.44 ± 3.24	29.92 ± 6.63
	Bachelor degree	194	26.62 ± 5.40	17.96 ± 3.57	31.23 ± 4.89
	Master degree or above	6	28.17 ± 2.40	18.67 ± 2.07	31.67 ± 4.80

Teaching experience	Yes	186	26.37 ± 5.49^a^	17.75 ± 3.69^b^	31.37 ± 4.90
	No	39	28.05 ± 3.39	18.74 ± 2.24	29.74 ± 5.87

Scientific research experience	Yes	16	26.75 ± 6.01	19.06 ± 1.61	31.44 ± 4.50
	No	209	26.65 ± 5.17	17.83 ± 3.59^b^	31.06 ± 5.15

Understanding of nursing association standard relevant to department	Grasp	119	27.38 ± 5.90	18.23 ± 4.03	32.73 ± 3.12^c^
	Know well	64	26.70 ± 3.36	18.05 ± 2.43	30.25 ± 5.17
	Know	40	25.18 ± 3.98	16.93 ± 3.06	27.83 ± 7.31
	Have not heard of	2	12.00 ± 8.49^a^	16.00 ± 5.66	25.00 ± 7.07

Whether or not have received training on association standard	Yes	198	27.07 ± 4.89	18.12 ± 3.41	31.44 ± 4.76
	No	27	23.67 ± 6.55^a^	16.52 ± 3.94^b^	28.48 ± 6.70^c^

The degree of recognition of the nursing profession	Approve	210	26.91 ± 5.21	18.17 ± 3.44	31.37 ± 4.96
	Normal	15	23.13 ± 4.02^a^	14.47 ± 2.47^b^	27.13 ± 5.63^c^
	Disagree	—	—	—	—

^a,b,c^Indicate statistically significant differences compared to other groups (*p* < 0.05).

**Table 4:** Assignment methods of argument variables.

Argument variables	Assignment methods
Gender	Male = 1, female = 2
Teaching experience	Yes = 1, no = 2
Scientific research experience	Yes = 1, no = 2
Understanding of nursing association standard relevant to department	Grasp = 1, know well = 2, know = 3, have not heard of = 4
Whether or not have received training on association standard	Yes = 1, no = 2
The degree of recognition of the nursing profession	Approve = 1, normal = 2, disagree = 3
Cognition of association standard	Plug in the original value
Organizational atmosphere	Plug in the original value

**Table 5:** Multivariate linear regression analysis of relevant influencing factors of association standard evidence-based nursing practice.

		*b*	SB	*t*	*p*
Cognition^a^	(Constant)	6.698	2.978	2.249	0.025
	Organizational atmosphere	0.179	0.020	8.770	< 0.001

Belief^b^	(Constant)	10.228	2.638	3.876	< 0.001
	Scientific research experience	−1.613	0.793	−2.033	0.043
	Organizational atmosphere	0.103	0.014	7.264	< 0.001

Behavior^c^	(Constant)	16.406	3.385	4.847	< 0.001
	Understanding of nursing association standard relevant to department	−1.386	0.385	−3.599	< 0.001
	Cognition of association standard	0.131	0.065	2.019	0.045
	Organizational atmosphere	0.123	0.024	5.242	< 0.001

^a^ *R* ^2^ = 0.323, adjusted *R*^2^ = 0.311, *F* = 26.232, *p* < 0.001.

^b^ *R* ^2^ = 0.273, adjusted *R*^2^ = 0.256, *F* = 16.455, *p* < 0.001.

^c^ *R* ^2^ = 0.343, adjusted *R*^2^ = 0.328, *F* = 22.859, *p* < 0.001.

## Data Availability

All data supporting the findings of this study are available within the paper. The datasets used and/or analyzed during the current study are available from the corresponding author on reasonable request.
